# Supplementary material for: Culture Media Composition Influences the Antibacterial Effect of Silver, Cupric, and Zinc Ions against Pseudomonas aeruginosa
Source: Biomolecules. 2022 Jul 9;12(7):963. doi: 10.3390/biom12070963 (PMC9312735; doi:10.3390/biom12070963)
Supplement: Supplementary file 1 [file biomolecules-12-00963-s001.zip › biomolecules-1785436-supplementary.pdf]

Supplementary materials

Culture media composition influences the antibacterial effect of silver, cupric, and zinc ions against *Pseudomonas aeruginosa*

Justyna Rewak-Soroczynska[1], Agata Dorotkiewicz-Jach[2]*, Zuzanna Drulis-Kawa[2], Rafal J. Wiglusz[1]*

[1]Institute of Low Temperature and Structure Research, Polish Academy of Sciences, Okolna 2, 50-422 Wroclaw, Poland; j.rewak@intibs.pl
[2]Department of Pathogen Biology and Immunology, University of Wroclaw, Przybyszewskiego 63/77, 51-148 Wroclaw, Poland; zuzanna.drulis-kawa@uwr.edu.pl

*Correspondence: agata.dorotkiewicz-jach@uwr.edu.pl (A.D.-J.); r.wiglusz@intibs.pl (R.J.W.)

Abstract: Different metals, such as silver (Ag), copper (Cu), and zinc (Zn), have been broadly investigated as free and doped elements of materials used both in medicine and everyday life due to their broad spectrum of antibacterial activity. Although the antibacterial action of those metals and their ions is well known and studied, the main problem remains in the standardization of experimental procedures to determine the antimicrobial activity as bacteriological media composition might significantly influence the outcome. The presented study aimed to evaluate the appropriability of different culture media (four nutritionally rich and four minimal) in the testing of the antibacterial activity of Ag^{+}, Cu^{2+}, and Zn^{2+} ions against *Pseudomonas aeruginosa.* Our investigation revealed the influence of medium ingredients and the presence of phosphates which significantly reduced the activity of tested metal ions. Moreover, the precipitate formation and decrease of pH in the minimal media were additionally observed. It was assumed that the most favorable medium for metal ions activity testing was Luria-Bertani complex medium and MOPS minimal medium.

Keywords: Bacterial culture media, Metal ions, Antibacterial activity, *Pseudomonas aeruginosa*

Table S1. The composition of minimal media used in the study.

	MOPS	M9	M63	Davis
Carbon	Glucose – $2{\cdot}10^{-2}$ M	Glucose – $2.2{\cdot}10^{-2}$ M	Glucose -$1.1{\cdot}10^{-2}$ M	Glucose – $5.5{\cdot}10^{-2}$ M
Fe^{2+}	$FeSO_4 \cdot 7\ H_2O$ –$1{\cdot}10^{-5}$ M	-	$FeSO_4 \cdot 7\ H_2O$ – $1.8{\cdot}10^{-6}$ M	-
Mg^{2+}	$Mg(NO_3)_2 \cdot 6\ H_2O$ – 5,2$\cdot10^{-4}$ M	$MgSO_4 \cdot 7H_2O$ – $2x10^{-3}$ M	$MgSO_4 \cdot 7\ H_2O$ – $1{\cdot}10^{-3}$ M	$MgSO_4 \cdot 7\ H_2O$ – $4{\cdot}10^{-4}$ M
NH^{4+}	NH_4Cl – 9,52$\cdot10^{-3}$ M	NH_4Cl – $1.97{\cdot}10^{-2}$ M	$(NH_4)_2SO_4$ – $1.5{\cdot}10^{-2}$ M	$(NH_4)_2SO_4$ – $5{\cdot}10^{-2}$ M
Ca^{2+}	$CaCl_2$ – $5{\cdot}10^{-7}$ M	$CaCl_2$ – $1{\cdot}10^{-4}$ M	-	-
PO_4^{3-}	K_2HPO_4 – $1.32x10^{-3}$ M	$Na_2HPO_4 \cdot 7H_2O$ – $4.8{\cdot}10^{-2}$ M<br>KH_2PO_4 – $2.2{\cdot}10^{-2}$ M	KH_2PO_4 – $1{\cdot}10^{-1}$ M	K_2HPO_4 – $2.68{\cdot}10^{-1}$ M<br>KH_2PO_4 – $9.8{\cdot}10^{-2}$ M
Other ingredients	MOPS – $4{\cdot}10^{-2}$ M<br>Tricine[N-Tris (Hydroxymethyl) Methylglycine] – $4x10^{-3}$ M<br>K_2SO_4 – $2.9{\cdot}10^{-4}$ M<br>NaCl – $5{\cdot}10^{-2}$ M<br>$(NH_4)_6Mo_7O_{24}$ - $3{\cdot}10^{-9}$ M<br>HBO_3 – $4{\cdot}10^{-7}$ M<br>$Co(NO_3)_2 \cdot 6\ H_2O$ – $3{\cdot}10^{-8}$ M<br>$CuSO_4$ x 5 H_2O – $1{\cdot}10^{-8}$ M<br>$Mn(NO_3)_2 \cdot H_2O$ – $8{\cdot}10^{-8}$M<br>$ZnSO_4 \cdot 7\ H_2O$ – $1{\cdot}10^{-8}$ M	NaCl – $8.6{\cdot}10^{-3}$ M	-	Sodium citrate – $1.29{\cdot}10^{-2}$ M
Reference	[1]	[2]	[2]	[3]

Table S2 Precipitation occurring after mixing the particular minimal media components with 1 mg/ml of metal cations (Ag^{+}, Cu^{2+}, Zn^{2+}).

	Ag^{+}	Cu^{2+}	Zn^{2+}
MOPS medium	++	+	+
MOPS stock	+ +	+	-
• MOPS	-	-	-
• Tricine	-	-	-
• $FeSO_4$	-	-	-
• NH_4Cl	+	-	-
• $Mg(NO_3)_2$	-	-	-
• NaCl	+	-	-
Glucose	-	-	-
K_2SO_4	-	-	-
K_2HPO_4	+	-	+
Davis medium	++	++	++
Davis salts	++	++	+
• K_2HPO_4	++	++	++
• KH_2PO_4	++	+	-
• $(NH_4)_2SO_4$	-	-	-
• Sodium citrate	+	-	-
$MgSO_4$ x 7 H_2O	-	-	-
Glucose	-	-	-
M9 medium	++	++	++
M9 salts	++	++	++
• Na_2HPO_4	++	++	+
• KH_2PO_4	++	+	-
• NaCl	++	-	-
• NH_4Cl	++	-	-
Glucose	-	-	-
$MgSO_4$ x 7 H_2O	+	-	-
$CaCl_2$	+	-	-
M63 medium	++	++	++
KH_2PO_4	++	+	-
$(NH_4)_2SO_4$	-	-	-
$MgSO_4$ x 7 H_2O	-	-	-
Glucose	-	-	-

Legend: ++ heavy precipitation, + mild precipitation, - no precipitation; color of the cells describe the color of obtained precipitate. Brownish and purplish color obtained for Ag^{+} was observed after exposing the obtained precipitate to the day light.

Table S3 Significant differences ($p<0.05$) calculated for the results presented in Figure 3 (1- $p<0.05$; 0 – $p\geq0.05$). The statistical analysis was performed using one-way-ANOVA and the Levene test, followed by the Tukey test in the OriginPro 2019b (OriginLab Corporation) software.

pH values		LB PAO1	LB ATCC 27853	MOPS PAO1	MOPS ATCC 27853
5.5	5	0	0	0	0
6	5	0	0	1	0
6	5.5	0	0	1	0
6.5	5	0	0	1	1
6.5	5.5	0	0	1	1
6.5	6	0	0	1	1
7	5	0	0	1	1
7	5.5	0	0	1	1
7	6	0	0	1	1
7	6.5	0	0	0	0
7.5	5	0	0	1	1
7.5	5.5	0	0	1	1
7.5	6	0	0	1	1
7.5	6.5	0	0	0	0
7.5	7	0	0	0	0
8	5	0	0	1	1
8	5.5	0	0	1	1
8	6	0	1	1	1
8	6.5	0	0	1	1
8	7	0	0	1	1
8	7.5	0	0	1	1

Correlation between medium composition and MIC values of tested ions

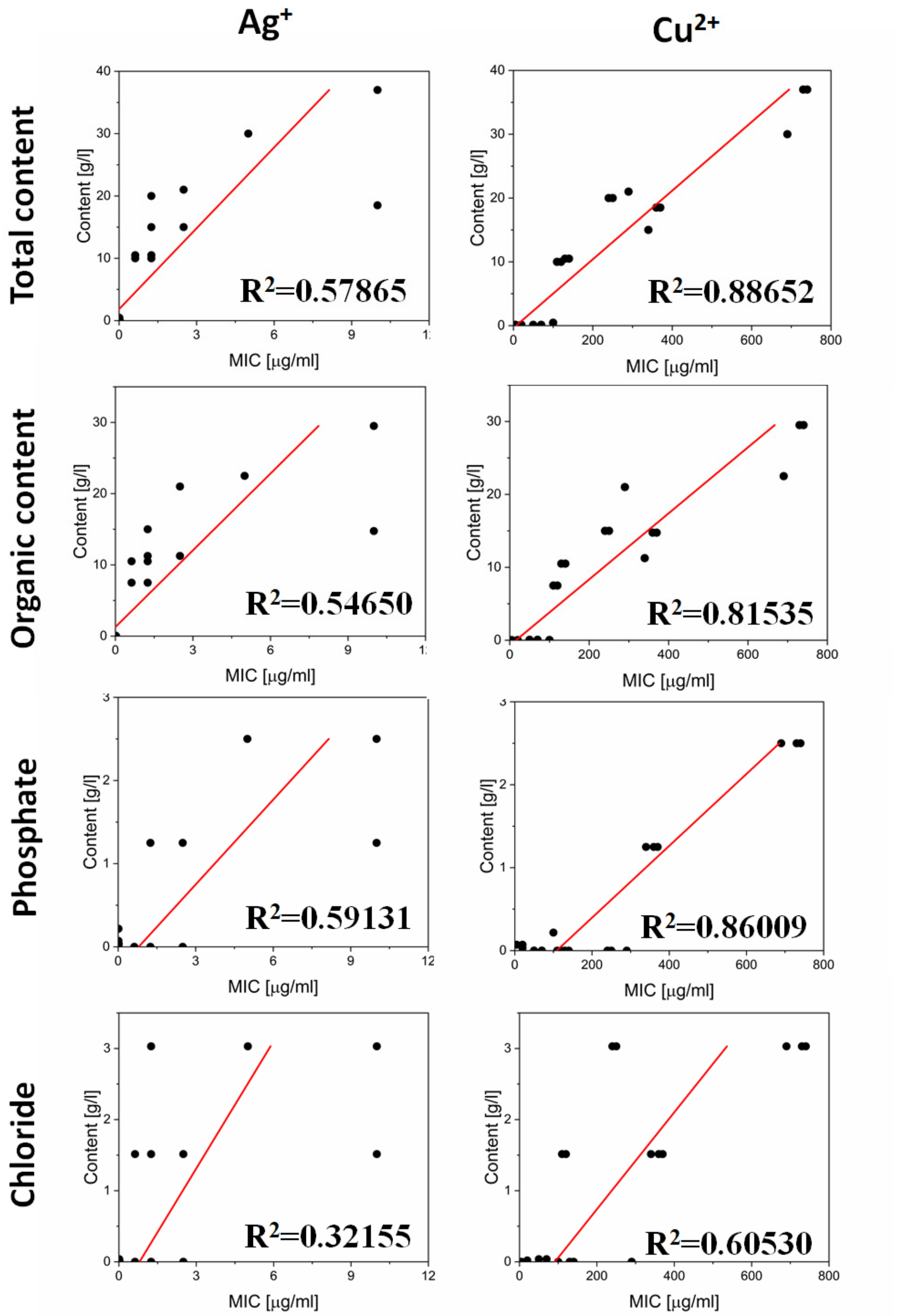

Figure S1. The correlation between the content of media components (total, organic, phosphate and chloride) and MIC values determined for *P. aeruginosa* PAO1 and ATCC 27853 strains.

Table S4. Characteristics of antibiotics susceptibility of *P. aeruginosa* strains [4].

P. aeruginosa	Source of strain		Antibiotics															
			**TIC**	**PIP**	**CAZ**	**GM**	**TOB**	**TIM**	**TZP**	**FEP**	**CFP**	**CTX**	**ME**	**IPM**	**ATM**	**AK**	**NET**	**CIP**
ATCC 27853	reference strain	**SENSITIVE**	-	-	-	-	-	-	-	-	-	-	-	-	-	-	-	-
PA01	reference strain	**SENSITIVE**	-	-	-	-	-	-	-	-	-	-	-	-	-	-	-	-
15/3	epiglottis	**SENSITIVE**	-	-	-	-	-	-	-	-	-	-	-	-	-	-	-	-
82/3	bronchia	**SENSITIVE**	-	-	-	-	-	-	-	-	-	-	-	-	-	-	-	-
9/5	trachea	**MDR**	R	R	R	-	-	R	R	-	R	R	R	R	R	-	-	-
14/3	intubation tube	**MDR**	R	-	-	R	R	R	-	-	R	R	-	R	-	-	-	R

(-) lack of resistance R-resistance according to EUCAST Clinical breakpoints - breakpoints and guidance (https://www.eucast.org/clinical_breakpoints/) ticarcillin (TIC) 75 mg, piperacillin (PIP) 100 mg, ceftazidime (CAZ) 30 mg, gentamicin (GM) 10 mg, tobramycin (TOB) 10 mg, ticarcillin/clavulanic acid (TIM) 75/10 mg, piperacillin/tazobactam (TZP) 100/10 mg, cefepime (FEP) 30 mg, cefoperazone (CFP) 75 mg, cefotaxime (CTX) 30 mg, imipenem (IPM) 10 mg, meropenem (MEM) 10 mg, aztreonam (ATM) 30 mg, amikacin (AK) 30 mg, netilmicin (NET) 30 mg, ciprofloxacin (CIP) 5 mg (Becton Dickinson and Company, Cockeysville, MD, USA)

References

1. LaBauve, A.E.; Wargo, W.J. Growth and Laboratory Maintenance of *Pseudomonas aeruginosa*. *Curr. Protoc. Microbiol.* **2012**. https://doi.org/10.1002/9780471729259.mc06e01s25.
2. Coffey, B.; Anderson, G. Biofilm Formation in the 96-Well Microtiter Plate. In *Pseudomonas Methods and Protocols*; Filloux, A., Ramos, J.-M., Eds.; Humana Press: New York, NY, USA, 2014; p. 634.
3. Atlas, R.M. *Handbook of Microbiological Media*; 3rd ed.; CRC Press: Boca Raton, FL, USA, 2004. https://doi.org/10.1201/9781420039726.
4. Sobierajska, P.; Dorotkiewicz-Jach, A.; Zawisza, K.; Okal, J.; Olszak, T.; Drulis-Kawa, Z.; Wiglusz, R.J. Preparation and Antimicrobial Activity of the Porous Hydroxyapatite Nanoceramics. *J. Alloys Compd.* **2018**, *748*, 179–187. https://doi.org/10.1016/j.jallcom.2018.03.162.
